# Febuxostat therapy improved the outcomes of cardiorenal syndrome rodent through alleviating xanthine oxidase-induced oxidative stress and mitochondrial dysfunction

**DOI:** 10.7150/ijbs.99194

**Published:** 2025-02-10

**Authors:** Chih-Chao Yang, Ya Yue, Yi-Ling Chen, John Y. Chiang, Yi-Ting Wang, Chi-Ruei Huang, Ben-Chung Cheng, Tsuen-Wei Hsu, Hon-Kan Yip

**Affiliations:** 1 Division of Nephrology, Department of Internal Medicine, Kaohsiung Chang Gung Memorial Hospital and Chang Gung University College of Medicine, Kaohsiung 833401, Taiwan, R.O.C.; 2 The First Hospital of Guangzhou Medical University, Guangzhou, 510120, China, P. R. C.; 3 Institute for Translational Research in Biomedicine, Kaohsiung Chang Gung Memorial Hospital Kaohsiung 833401, Taiwan, R. O. C.; 4 Division of Cardiology, Department of Internal Medicine, Kaohsiung Chang Gung Memorial Hospital and Chang Gung University College of Medicine, Kaohsiung 833401, Taiwan, R. O. C.; 5 Department of Computer Science and Engineering, National Sun Yat-Sen University, Kaohsiung 804201, Taiwan, R. O. C.; 6 Department of Healthcare Administration and Medical Informatics, Kaohsiung Medical University, Kaohsiung 807378, Taiwan, R. O. C.; 7 Center for Shockwave Medicine and Tissue Engineering, Kaohsiung Chang Gung Memorial Hospital Kaohsiung 833401, Taiwan, R. O. C.; 8 Department of Medical Research, China Medical University Hospital, China Medical University, Taichung 404333, Taiwan, R. O. C.; 9 School of Medicine, College of Medicine, Chang Gung University, Taoyuan 333323, Taiwan, R. O. C.

**Keywords:** xanthine oxidase, oxidative stress, mitochondrial biogenesis, inflammation, apoptosis

## Abstract

**Background:** We tested the hypothesis that febuxostat (Feb) therapy effectively protected cardiorenal syndrome (CRS) rats via repressing the xanthine-oxidase (XO)-caused oxidative stress.

**Methods and Results:** Cellular levels of apoptosis/oxidative stress/mitochondrial-membrane potential were higher in p-Cresol treated-NRK-52E cells than in control group that were reversed by Feb treatment or silencing XO gene (all *P*<0.001). Pilot study demonstrated that: XO activity was significantly increased in CRS than in SC group; a significant negative correlation between XO activity and left ventricular ejection fraction (LVEF) (%); a significant positive correlation between XO activity and BNP/BUN/creatinine/proteinuria levels (all *P*<0.01). Male-adult SD-rats were classified into groups 1(sham-control)/2 (CRS)/3 [CRS+Feb (10mg/kg/day)]/4 [CRS+Feb (30mg/kg/day)]. By day-63, the survival rate was significantly lower in group 2 than in other groups (*P*=0.029), and circulatory levels of FGF23/BNP/XO-activity BUN/creatinine/proteinuria and renal-artery resistance were highest in group 2/lowest in group 1/significantly lower in group 4 than in group 3, whereas the LVEF exhibited an opposite pattern of XO among the groups (all *P*<0.0001). Cellular levels of fibrosis/XO/H_2_DCFDA/CD68/CHAC1, and protein expressions of oxidative-stress (NOX-2/NOX-4/XO)/inflammatory (NF-κB/IL-1β)/fibrotic (Smad3/TFG-β)/apoptotic (CHAC1/2)/mitochondrial-damaged (p-DRP1) biomarkers in kidney/heart tissues displayed a similar pattern of XO (all *P*<0.0001).

**Conclusion:** Feb therapy improved cardiorenal function and prognostic outcome in CRS rats.

## Introduction

Cardiorenal syndrome (CRS) is a complex pathophysiological disorder that is a composite of a globally well-known interaction between heart disease and kidney disease, in which acute or chronic damage of one organ can cause acute or chronic dysfunction of another organ 1-3. Thus, CRS is two sites of the same coin, i.e., one side called “heart” and the other side called “kidney”, that mutually affect each other, such as hemodynamic factors of low cardiac output, fluid overload, heart failure, overproduction of uremic toxic substance, oxidative stress and inflammation, all accounting for the pathogenesis and poor prognostic contributors of CRS 3. It is well recognized that the mortality, morbidity and unfavorable long-term outcome are remarkably higher in CRS than that of merely one organ disorder, i.e., either cardiovascular disease (CVD) or chronic kidney disease (CKD). The common risk factors of CRS include hypertension, diabetes mellitus, elderly age, oxidative stress-related endothelial dysfunction in aging, and prior history of heart or renal failure 2, 4-6. Therefore, the key to improve CRS outcome is to interrupt the vicious cycle through the therapies with dual beneficial effects on both heart and kidney protection.

Xanthine oxidoreductase (XOR), including xanthine dehydrogenase (XDH) and xanthine oxidase (XO), is a complex enzyme that catalyzes the oxidative reaction from hypoxanthine to xanthine, resulting in generation of uric acid 7. XOR has been identified as a crucial source of reactive oxygen species (ROS) in a variety of pathophysiological circumstances 8-11. In addition, ROS generated by XO also participates in intermittent hypoxia-evoked NOX activation via calcium-dependent protein kinase C stimulation 12. Uric acid, the final product of metabolism of purine by XO, serves as a pro-oxidant inside the cell where it activates NADPH oxidase enzyme to upregulate intracellular level of oxidative stress, mitochondrial injury, and ATP depletion 13. Besides being involved in production of uric acid, XO can also augment oxidative stress and reduce NO production and bioavailability, inducing further endothelial dysfunction, inflammation, tissue hypoxia and ROS production, ultimately resulting in renal failure through increased renal interstitial fibrosis 14. In myocardium, XO is localized in the capillary endothelial cells. Experimental evidence has suggested that endothelial cells can express XO and produce a marked elevation in XO levels when exposed to ischemia or hypoxia 15. Because XO in failing myocardium is elevated, various studies reveal that XO participates in oxidative stress and concomitant overproduction of uric acid in CVD, especially in the scenario of heart failure (HF) 16, 17. It is reported that the activity of endothelial bound XO is increased by more than 200% in patients with HF 9. Another study shows that XO expression and activity are increased in the remote myocardium of mice after myocardial infarction in association with increased oxidative stress 18. Additionally, myocardial XO expression has been shown to be increased in patients with HF, indicating that XO activity may contribute to abnormal energy metabolism in human cardiomyopathy 17.

Numerous studies have identified XO is commonly present in kidney 19 and myocardium 18, 20. Additionally, febuxostat (Feb), a xanthine oxidase inhibitor, effectively alleviated the gouty arthritis though lowering serum uric acid hyperuricemia 21 as a subsequence of ensuring a renal-protective effect and suppressing the generation of intracellular reactive ROS in endothelial cells 13. Furthermore, a placebo controlled randomized trial showed that Feb slowed the decline in eGFR in CKD stages 3 and 4 compared to placebo 22.

According to the aforementioned issues, we design a preclinical study to test the hypothesis that Feb might be an innovative drug for protecting the heart and kidney organs against CRS damage.

## Materials and methods

### Ethical issues

All animal procedures were approved by the Institute of Animal Care and Use Committee at Kaohsiung Chang Gung Memorial Hospital (Affidavit of Approval of Animal Use Protocol No. 2018122026) and performed in accordance with the Guide for the Care and Use of Laboratory Animals. Animals were housed in an Association for Assessment and Accreditation of Laboratory Animal Care International (AAALAC; Frederick, MD, USA)-approved animal facility in our hospital with controlled temperature and light cycles (24 °C and 12/12 light cycle).

### Cell grouping for *in vitro* studies

For the purpose of the study, the rat podocyte cell line which was friendly gifted from Dr. Yang who works at Institute for Translational Research in Biomedicine, Kaohsiung Chang Gung Memorial Hospital, was grown at 37 °C under 5% CO_2_ in culture dishes containing Dulbecco's modified Eagle's medium 11965-084, GIBCO) supplemented with 10% fetal bovine serum, 1% Non-Essential Amino Acids (NEAA), 100 μg/ml streptomycin, and 100 units/ml penicillin. The p-Cresol was purchased from Sigma and utilized to treat the cells with different concentrations at 48h, respectively.

### Transfection of siRNA with plasmid

Transient transfection of NRK-52E cells with siRNA was performed with Lipofectamine™ RNAiMAX Transfection Reagent (Invitrogen, Life technologies, Carlsbad, CA, USA) according to the manufacturer's instructions but with slight modifications. Cells were replated 24hrs before transfection at a density of 1 x 10^6^ cells in 7 ml of fresh culture medium in a 10-cm plastic dish. For use in transfection, Lipofectamine™ RNAiMAX Transfection Reagent was incubated with 100 pmol of indicated siRNA at room temperature for 15 minutes. Cells were incubated with siRNA complex at 37°C in a humidified atmosphere of 5% CO_2_ before being harvested. Western blotting and RT-PCR technique were performed to verify the efficiency of gene-silencing.

### MTT assay for cell viability

The procedure and protocol have been reported by our previous study 23. Briefly, cell growth was determined by the MTT assay. About 2 x 10^3^ cells in 100 µL of medium were seeded into wells of a 96-well plate and incubated for the indicated duration. At the designed time points (i.e., at 24, 48 and 72hrs) of cell incubation, 200 uL MTT solution (500 ug/mL) was added into each well. After incubation, the purple crystal sediment was dissolved in 100 uL DMSO and read out at 540 nm in an ELISA reader. The absorbance value was used to represent the cell number.

### Flow cytometric analysis for apoptosis detection in NRK-52E

The percentages of viable and apoptotic cells were determined by flow cytometry using double staining of annexin V and propidium iodide (PI). The early phase of apoptosis was defined as annexin V+/PI-, whereas the late phase of apoptosis was defined as annexin V+/PI+.

### Measuring the levels of ROS in culturing NRK-52E cells

Total intracellular ROS were measured by the H_2_DCF-DA oxidation method. Briefly, the cells were incubated in HEPES buffer supplemented with H_2_DCF-DA for 30 minutes. H_2_DCF-DA is a cell-permeable probe that is oxidized by intracellular ROS to generate fluorescent DCF. The fluorescent DCF was detected by immunofluorescent microscope.

### CRS model by 5/6 subtotal nephrectomy for induction of CKD, and doxorubicin (DOX) of induction of dilated cardiomyopathy (DCM)

The procedure and protocol of CRS induction have been described in our previous report 23. In detail, pathogen free, adult male SD rats, weighing 275-325 g (Charles River Technology, BioLASCO Taiwan Co., Ltd., Taiwan), age of 8-12 weeks, after 7-day adaption period, were randomly allocated to different groups. The animal model of CRS was defined as: (1) DCM induced for first 20 days, followed by (2) CKD model 2 weeks (total need 35 days, equal to day 0 of CRS). DCM was first induced, followed by CKD induction in the same animal to finally became as the CRS model.

The animals were then randomly divided into four groups, i.e., group 1 [sham-operated control (SC)], group 2 [CRS (i.e., DCM + CKD)], group 3 [CRS + Feb (10 mg/kg/day since day 35 after DCM induction) by oral administration)] and group 4 [CRS + Feb (30 mg/kg/day since day 35 after DCM induction) by oral administration]. Animals in each group were euthanized by day 63 after DCM induction (i.e., total four week's Feb treatment).

### Animal model of dilated cardiomyopathy (DCM)

The procedure and protocol as well as the optimal dosage of doxorubicin to induce DCM have been reported in our previous study in detail 23. Briefly, each animal in DCM group was treated by an accumulated dosage of 7.5 mg/kg doxorubicin (DOX) [intraperitoneal administration once at each 5-day's interval within 20 days, i.e., at 4 separated time points (refer to Supplementary Fig. 1)]. Transthoracic echocardiography specific for animal study was used for assessing the successful conduction of a rat DCM model in the present study.

### Animal model of CKD

The protocol and procedure of creation of CKD were based on our previous report in detail 23. Briefly, by day 20 after DCM induction, all animals were anesthetized with 2.0% inhalational isoflurane for midline laparotomies. The SC group received laparotomy only, followed by closure of the muscle and skin layers, while CKD induction was performed in the remaining groups. CKD was conducted by right nephrectomy plus arterial ligation of upper and middle thirds of blood supplies to the left kidney, thereby creating a 5/6 nephrectomy model with limited renal function (refer to Supplementary Fig. 1). In this way, combination of DCM and CKD was defined as the animal model of CRS in rat for the purpose of the current study.

### Left ventricular function evaluated by echocardiography

The procedure and protocol for transthoracic echocardiography were based on our previous report 23. In detail, transthoracic echocardiography (Vevo 2100, Visualsonics Toronto, Ontario, Canada.) was performed in each group at day 0 prior to DCM induction and days 35 and 60 after by an animal cardiologist blind to the experimental design. M-mode standard two-dimensional left parasternal/long axis echocardiographic examination was conducted. The LV internal dimensions [i.e., LV end-systolic diameter (LVESd) and LV end-diastolic diameter (LVEDd)] were measured at the mitral valve and papillary levels of the left ventricle, according to the American Society of Echocardiography (Morrisville, NC) leading-edge method using at least three consecutive cardiac cycles. The LVEF was calculated as follows: LVEF (%) = [(LVEDd^3^ - LVESd^3^)/LVEDd^3^] × 100%.

### To assess the serial changes of renal artery resistive index (RARI)

The RARI is an essential and reliable factor for assessing the renal arterial resistance in various kidney diseases, such as CKD. By using the ultrasound machine (Vevo 2100, Visualsonics), an expert animal ultrasound specialist measured two parameters of renal artery, including (1) peak systolic blood velocity (PSV) and (2) lowest diastolic blood velocity (LDV) by day 0 prior to and by days, 35 and 63 after DCM induction. Thus, the calculation formula was defined as RARI = (PSV-LDV)/PSV.

### Serially collected the blood samples for assessment of renal function

In the present study, serial blood samples for measuring the circulating levels of blood urine nitrogen (BUN) and creatinine were drawn at day 0 prior to and by days 35, 42 and 63 after DCM induction. An amount of 3 ml peripheral blood from tail vein was collected at each time point, followed by adequate fluid supply, i.e., 6 mL normal saline was intraperitoneally administered each time after blood sampling.

### Serially collected the 24h urine for assessment of the ratio of urine protein to urine creatinine (R^Up/Uc^)

The procedure and protocol have been reported in our previous studies 23, 24. Briefly, for the collection of 24hrs urine in individual study, each animal was put into a metabolic cage (DXL-D, space: 190 x 290 x 550 mm^3^, Suzhou Fengshi Laboratory Animal Equipment Co. Ltd., China) for 24hrs with free access to food and water. Urine in 24h was collected in all animals prior to and by days 35, 42 and 60 after CRS induction for determining the R^Up/Uc^.

### Histological study of fibrosis areas

The procedure and protocol were based on our previous reports 23, 24. In detail, Masson's trichrome was utilized for assessing fibrosis in kidney parenchyma and left ventricular (LV) myocardium. Three 4 µm thick serial sections of kidney and LV myocardium were prepared by Cryostat (Leica CM3050S). The integrated area (µm^2^) of fibrosis in each section was calculated using Image Tool 3 (IT3) image analysis software (University of Texas, Health Science Center, San Antonio, UTHSCSA; Image Tool for Windows, Version 3.0, USA). Three selected sections were quantified for each animal. Three randomly selected HPFs (100x) were analyzed in each section. After determining the number of pixels in each fibrotic area per HPF, the numbers of pixels obtained from the three HPFs were summated. The procedure was repeated in two other sections for each animal. The mean pixel number per HPF for each animal was then determined by summating all pixel numbers and dividing by 9. The mean integrated area (µm^2^) of fibrosis in kidney and in LV myocardium per HPF was obtained using a conversion factor of 19.24 (1 µm^2^ represented 19.24 pixels).

### Western blot assay

The procedure and protocol were based on our previous reports 23, 24. Briefly, equal amounts (50 μg) of protein extracts were loaded and separated by SDS-PAGE using acrylamide gradients. After electrophoresis, the separated proteins were transferred electrophoretically to a PVDF membrane (GE, UK). Nonspecific sites were blocked by incubation of the membrane in blocking buffer [5% nonfat dry milk in T-TBS (TBS containing 0.05% Tween 20)] overnight. The membranes were incubated with the indicated primary antibodies [phosphorylated (p)-AMP-activated protein kinase (p-AMPK) (1:1000, Cell Signaling AMPK (1:1000, Cell Signaling), peroxisome proliferator-activated receptor gamma coactivator 1-*alpha* (PGC-1α) (1:1000, Abcam), Sirtuin 1 (SIRT1) (1:1000, Abcam), copper zinc superoxide dismutase 1 (SOD1) (1:1000, Abcam), xanthine oxidase (XO) (1:1000, Abcam), p-mTOR (1:1000, Cell Signaling), mTOR (1:1000, Cell Signaling), Catalase (1:1000, Abcam), NOX2 (1:1000, Sigma), NOX4 (1:1000, Abcam), dynamin-related protein 1 (*DRP1*) (1:1000, Cell Signaling), ChaC glutathione specific gamma-glutamylcyclotransferase 1 (CHAC1) (1:1000, Proteintech), CHAC2 (1:1000, Proteintech), p-Smad3 (1:1000, Cell Signaling), transforming growth factor-β (TGF-β) (1:1000, Abcam), p-nuclear factor (NF)-κB (1:1000, Cell Signaling), interleukin IL-1β (1:1000, Cell Signaling)] for 1 hour at room temperature. Horseradish peroxidase-conjugated anti-rabbit immunoglobulin IgG (1:6000, Sigma) was used as a secondary antibody for one-hour incubation at room temperature. The washing procedure was repeated eight times within one hour. Immunoreactive bands were visualized by enhanced chemiluminescence (ECL; Amersham Biosciences, Amersham, UK) and exposed to Biomax L film (Kodak, Rochester, NY, USA). For the purpose of quantification, ECL signals were digitized using Labwork software (UVP, Waltham, MA, USA). Additionally, anti-rabbit IgG horseradish peroxidase-conjugated antibodies (1:2000, Cell Signaling Danvers, MA) were utilized. Furthermore, the results were normalized to beta-actin expression.

### Immunohistochemical (IHC) and Immunofluorescent (IF) staining

The procedure and protocol for IHC and IF staining have been described in our previous reports 23, 24. For IHC and IF staining, rehydrated paraffin sections were first treated with 3% H_2_O_2_ and incubated with Immuno-Block reagent (BioSB, Santa Barbara, CA, USA) for 30 minutes at room temperature. Sections were then incubated with primary antibodies specifically against Xanthine Oxidase (1:500, Abcam), CD68 (1:500, Abcam), Glutathione specific gamma-glutamylcyclotransferase 1 (CHAC1) (1:100, Proteintech), and CHAC2 (1:100, Proteintech), while sections incubated with the use of irrelevant antibodies served as controls. Three sections of kidney specimen and quadriceps muscle from each rat were analyzed. For quantification, three random chosen HPFs (200x or 400x for IHC and IF studies) were analyzed in each section. The mean number of positively stained cells per HPF for each animal was then determined by summation of all numbers divided by 9.

An IHC-based scoring system was adopted for semi-quantitative analysis of CHAC1 in the kidney as a percentage of positive cells in a blinded fashion (score of positively stained cells for these biomarkers as: 0 = negative staining; 1= <15%; 2 = 15-25%; 3 = 26-50%; 4 = 51-75%; 5= 76-100% per HPF).

### Statistical analysis

Quantitative data were expressed as mean ± SD. Statistical analysis was adequately performed by ANOVA followed by Bonferroni multiple-comparison post hoc test. Statistical analysis was performed using SPSS statistical software for Windows version 22 (SPSS for Windows, version 22; SPSS, IL, USA). A value of *P*<0.05 was considered as statistically significant.

## Results

### The impact of p-Cresol on oxidative stress generation and podocytes damage and the protective effect of Feb therapy (Figure 1)

First, we wanted to verify the impact of uremic toxic substance on damaging the glomeruli, and hence the podocytes were categorized into: (1) podocytes only, and (2) podocytes + p-Cresol (10, 50 and 200 µM). The *in vitro* results showed that as compared with the control group, the protein expressions of NOX-1, NOX-2, and XO, three indices of oxidative stress, and cellular level of γ-H2AX, an indicator of DNA damage, were notably progressively enhanced as the concentration of p-Cresol was progressively increased, whereas the protein expressions of ZO-1, synaptopodin and podocin, three indicators of podocyte components, exhibited an opposite pattern of oxidative stress.

Additionally, to elucidate the impact of Feb therapy on protecting the podocytes, these cells were categorized into: (1) podocytes only, (2) podocyte + p-Cresol (200 µM), (3) podocyte + p-Cresol (200 µM) + Feb (50, 100 µM) and (4) silencing XO gene (i.e., siRNA knockdown of XO gene) in podocyte + p-Cresol (200 µM), respectively, and the aforementioned parameters were analyzed again by following the same procedure. The results showed that all the parameters displayed an opposite pattern themselves among the groups in conditions of high dose of Feb (i.e., 100 µM) and silencing OX, suggesting that XO played a key role on oxidative stress and cell damage and Feb therapy offered a protective effect on podocytes.

### Flow cytometric analysis for assessment of impact of Feb treatment on cell viability and total intracellular and mitochondrial ROS undergoing p-Cresol stimulation (Figure 2)

In this *in vitro* study, the NRK-52E cells were classified into: (1) NRK-52E cells only, (2) NRK-52E cells + p-Cresol (200 µM), (3) NRK-52E cells + p-Cresol (200 µM) + Feb (100 µM) and (4) silencing XO gene in podocytes + p-Cresol (200 µM), respectively. The parameters of cell viability at 24, 48 and 72hrs, and total intracellular and mitochondrial ROS at 24h were measured, respectively. The result of flow cytometric analysis showed that as compared with control group, the cell viability at the time points of 24, 48 and 72hrs were significantly reduced in p-Cresol-treated (200 µM) NRK-52E cells that were significantly revised by Feb treatment or siRNA OX gene silencing in NRK-52E cells. Additionally, mean intensities of total intracellular (i.e., H_2_DCF-DA stain) and mitochondrial ROS (mitoSOX stain) expression displayed an opposite pattern of cell viability among the groups.

### Flow cytometric analysis for apoptosis detection and mitochondrial membrane potential for evaluating mitochondrial function (Figure 3)

For flow cytometric analysis, the cells were categorized into: (1) NRK-52E only, (2) NRK-52E + p-Cresol (200 µM), (3) NRK-52E + p-Cresol (200 µM) + Feb (100 µM) and (4) NRK-52E + p-Cresol (200 µM) + Feb (200 µM), respectively.

The results of flow cytometric analysis showed that, as compared to SC group, the early and late apoptosis were significantly increased in NRK-52E treated by p-Cresol that were significantly reversed by lower dose of Feb and furthermore significantly reversed by higher dose of Feb, whereas the mitochondrial membrane potential revealed an opposite pattern of apoptosis among the groups.

### Pilot studies were conducted to support our hypothesis that XO played a cardinal role and therapeutic benefits of febuxostat (Figures 4 and 5)

In the pilot studies, our missions were to elucidate several issues at day 42 after CRS induction, including the circulatory level of XO activity in CRS animals, the correlations between circulatory XO activity and circulatory creatinine, BUN and BNP levels, LVEF and the ratio of urine protein to urine creatinine as well as the XO activity in LV myocardium and kidney in CRS animals.

As were expected, circulatory levels of XO and brain natriuretic peptide (BNP), a pressure overload/heart failure biomarker, were significantly higher in CRS group than in SC group (Fig. 4). Additionally, there was a strong positive correlation between circulating levels of BNP and XO activity (Fig. 4). On the other hand, a significantly negative correlation between LVEF and circulatory XO activity was observed in this pilot study (Fig. 4).

Next, we assessed the correlations between XO activity and the renal dysfunction parameters in CRS rats by day 42 after DCM induction. The result demonstrated that the circulating levels of BUN and creatinine and the ratio of urine protein to urine creatinine (R^Up/Uc^) were significantly increased in CRS group than in SC group (Fig. 4). Additionally, there were significant positive correlations between XO activity and creatine, BUN and R^Up/Uc^ (Fig. 4).

**Figure 1:**
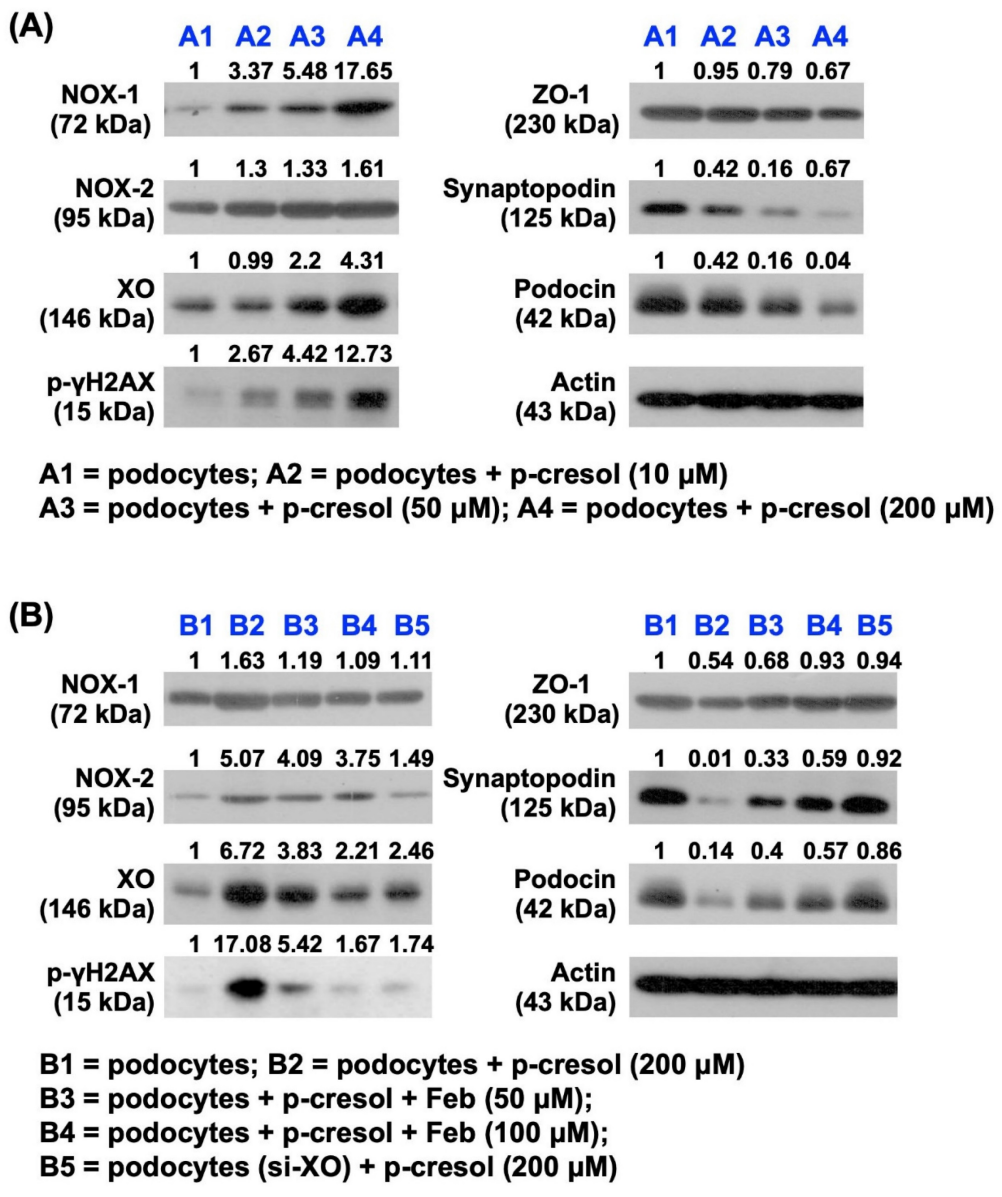
** The impact of p-Cresol on oxidative stress production and podocyte damage, and the protective effect of febuxostat treatment. A)** Protein expressions of NOX-1, NOX-2, OX and phosphorylated (p)-γ-H2AX were notably progressively upregulated as the concentration of p-Cresol was progressively increased (i.e., 0, 10, 50, 200 µM). On the other hand, the protein expressions of *ZO*-*1*, synaptopodin and podocin were remarkably progressively attenuated as the concentration of p-Cresol was progressively increased. **B)** Undergoing a fixed high dose of p-Cresol (200 µM) treatment, the protein expressions of NOX-1, NOX-2, XO and γ-H2AX were notably progressively suppressed as the concentration of Feb was increased (i.e., 0, 50, 100 µM), whereas the protein expressions of ZO-1, synaptopodin and podocin displayed an opposite manner of oxidative stress. Similarly, silencing the XO in the podocytes undergoing a fixed high dose of p-Cresol (200 µM) treatment also contributed a consistent impact as Feb treatment among the groups. n = 1 for each group; Feb = febuxostat; XO = xanthine oxidase; ZO-1 = Zonula occludens-1.

**Figure 2:**
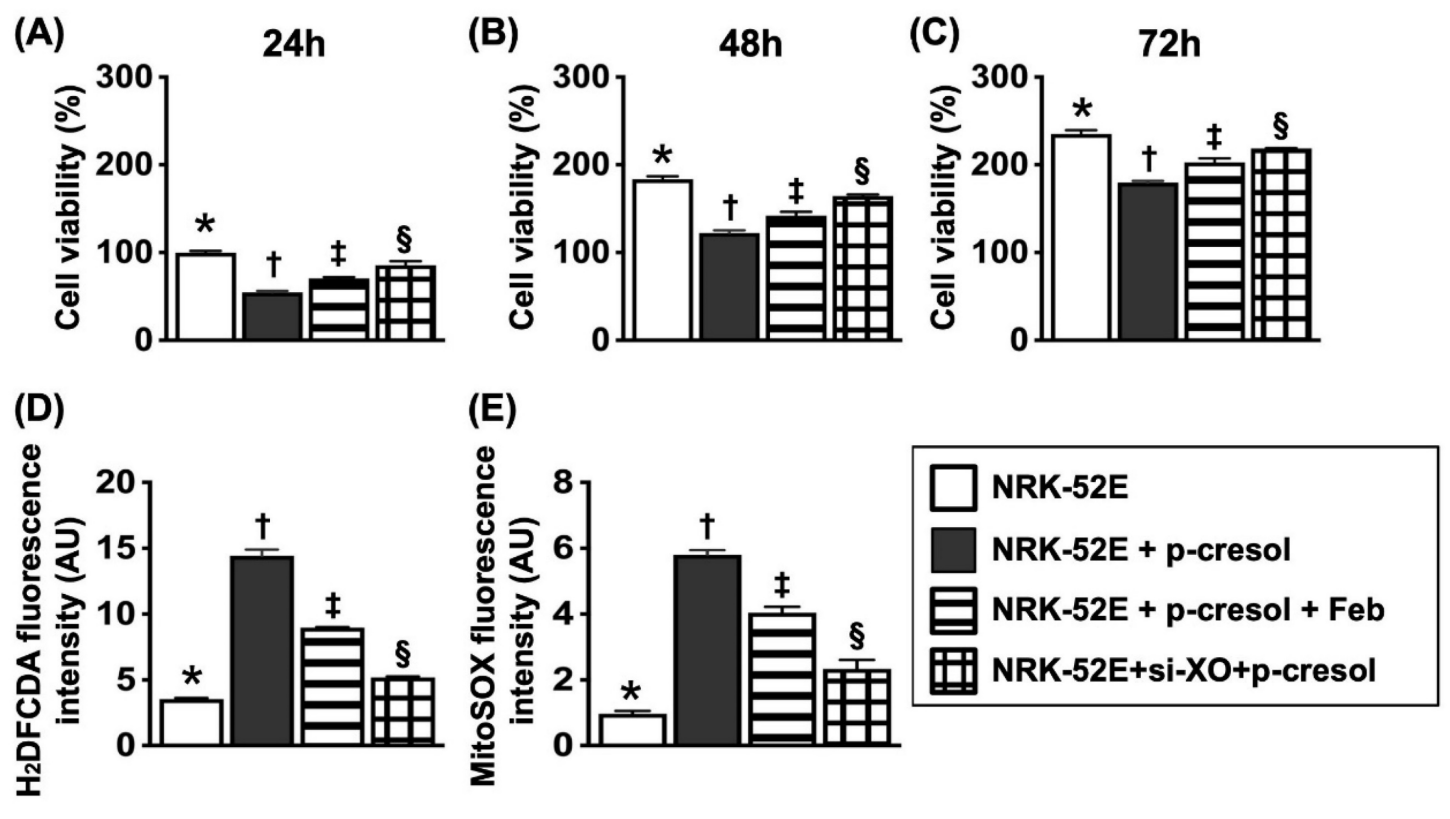
** Flow cytometric analysis for assessment of impact of febuxostat treatment on cell viability and total intracellular and mitochondrial ROS undergoing p-Cresol stimulation. A)** MTT assay for identification of NRK-52E cell viability at 24h, * vs. other groups with different symbols (†, ‡, §), *P*<0.0001. **B)** MTT assay for identification of NRK-52E cell viability at 48hrs, * vs. other groups with different symbols (†, ‡, §), p<0.0001. **C)** MTT assay for identification of NRK-52E cell viability at 72h, * vs. other groups with different symbols (†, ‡, §), *P*<0.0001. **D)** Flow cytometric analysis of mean fluorescent intensity of total intracellular ROS (i.e., by H_2_DCF-DA stain), * vs. other groups with different symbols (†, ‡, §), *P*<0.0001. **E)** Flow cytometric analysis of mean fluorescent intensity of mitochondrial ROS (i.e., by mitoSOX stain), * vs. other groups with different symbols (†, ‡, §), *P*<0.0001. All statistical analyses were performed by one-way ANOVA, followed by Bonferroni multiple comparison post hoc test (n=6 for each group). Symbols (*, †, ‡, §) indicate significance (at 0.05 level).

**Figure 3:**
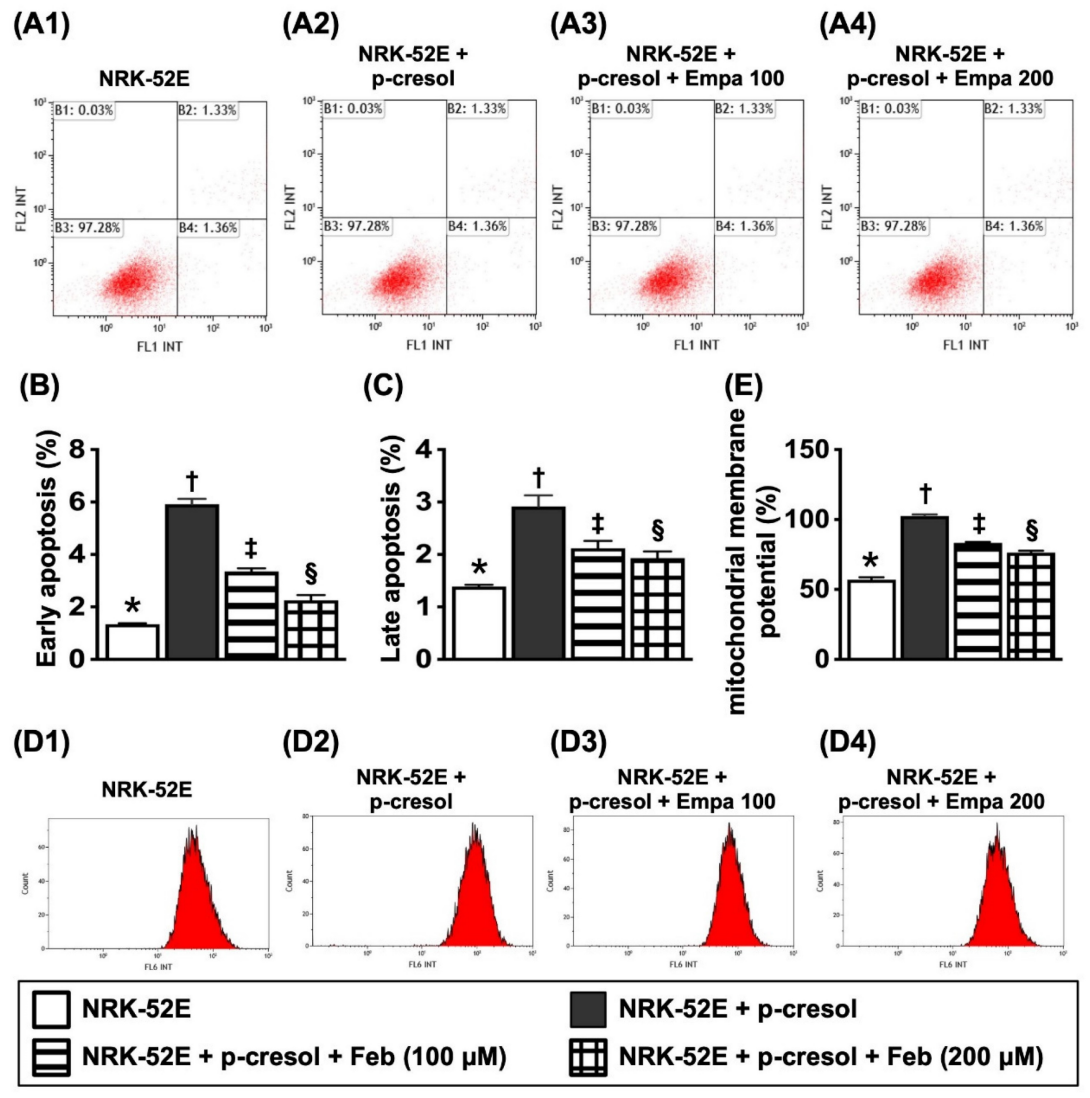
** Flow cytometric analysis for apoptosis detection and mitochondrial membrane potential for evaluating mitochondrial function. A1 to A4)** Illustrating the flow cytometric analysis for identification of early (V+/PI-) and late (V+/PI+) apoptosis. **B)** Analytical result of early apoptosis, * vs. other groups with different symbols (†, ‡, §), *P*<0.0001. **C)** Analytical result of late apoptosis, * vs. other groups with different symbols (†, ‡, §), *P*<0.0001. **D1-D4)** Flow cytometric analysis for identification of mitochondrial function (i.e., by MitoTracker deep red staining). **E)** Analytical results of mitochondrial membrane potential (MMP), * vs. other groups with different symbols (†, ‡, §), *P*<0.0001. All statistical analyses were performed by one-way ANOVA, followed by Bonferroni multiple comparison post hoc test (n=6 for each group). Symbols (*, †, ‡, §) indicate significance (at 0.05 level). XO = xanthine oxidase.

**Figure 4:**
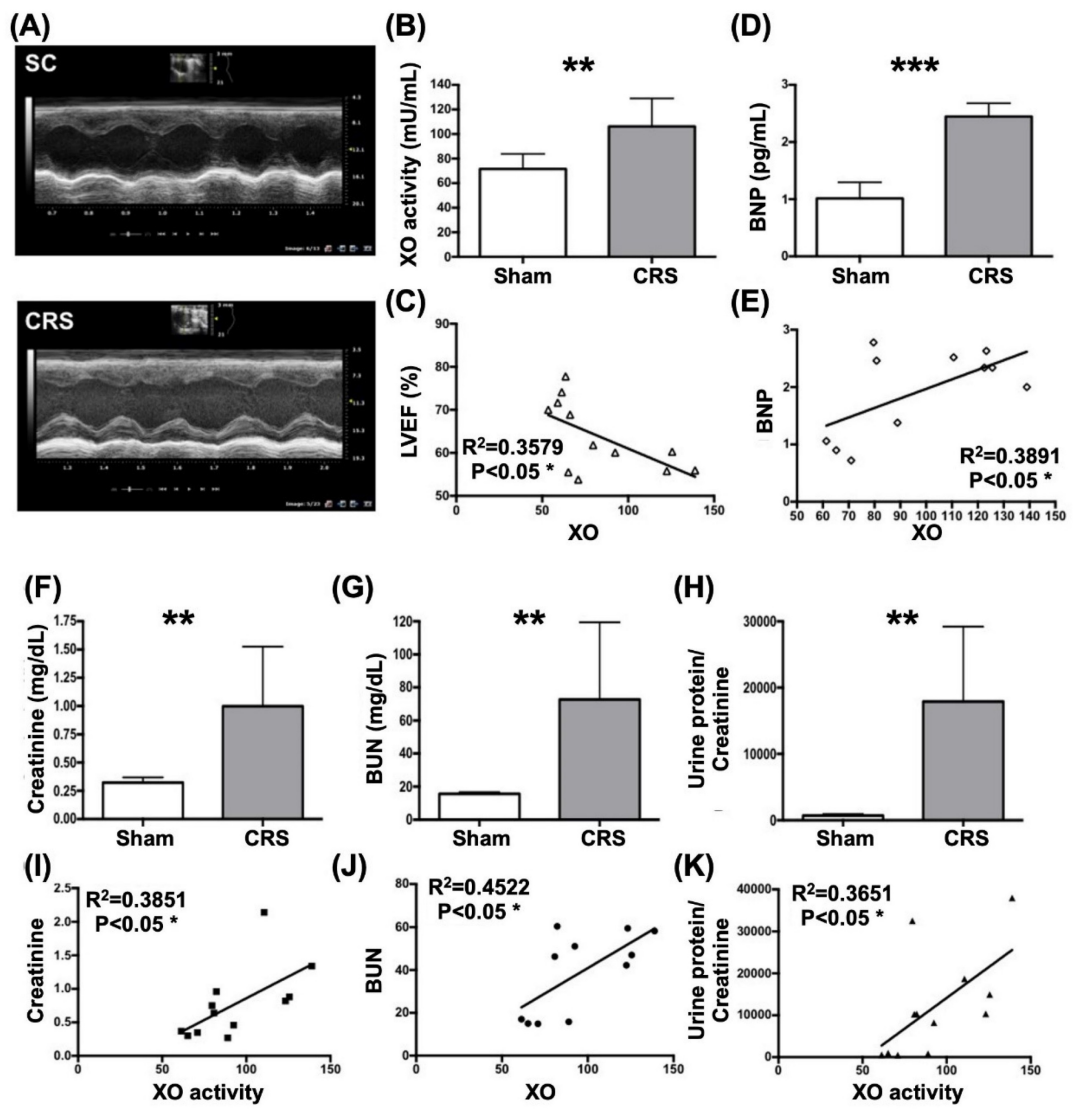
**XO activity remarkably elevated and significantly positively correlated to heart and renal dysfunction in rat CRS model by day 42 after CRS induction. A)** Illustrating the transthoracic echocardiographic imaging in SC group and CRS group, respectively. The M-mode showed that the left ventricular (LV) contractility notably reduced, whereas the chamber dilatation was notably increased in CRS animal as compared with SC, implicating LV remodeling developed in CRS animal. **B)** The XO activity was significantly increased in CRS than in SC group. **C)** Analytical result showed a significant negative correlation between XO activity and left ventricular ejection fraction (LVEF) (%). **D)** The circulatory level of BNP was significantly increased in CRS group than in that of SC group. **E)** Analytical result showed a significant positive correlation between circulatory level of BNP and XO activity. **F)** Circulating level of creatine level was significantly increased in CRS group than in SC group by day 42 after CRS induction. **G)** Circulating level of blood urea nitrogen (BUN) was significantly increased in CRS group than in SC group by day 42 after CRS induction. **H)** The ratio of urine protein to urine creatinine (R^Pu/Uc^) (C) was significantly elevated in CRS group as compared to that of the SC group. **I to K)** Analytical results showed there were significantly positive correlations between XO activity and creatine (I), BUN (J) and the R^Pu/Uc^ (K). The statistical analyses were calculated by t-test or Pearson's correlation. ** indicating *P*<0.001; *** indicating *P*<0.0001 (n=6-12/group). SC = sham control; CRS = cardiorenal syndrome; BNP = brain natriuretic peptide; XO = xanthine oxidase.

**Figure 5:**
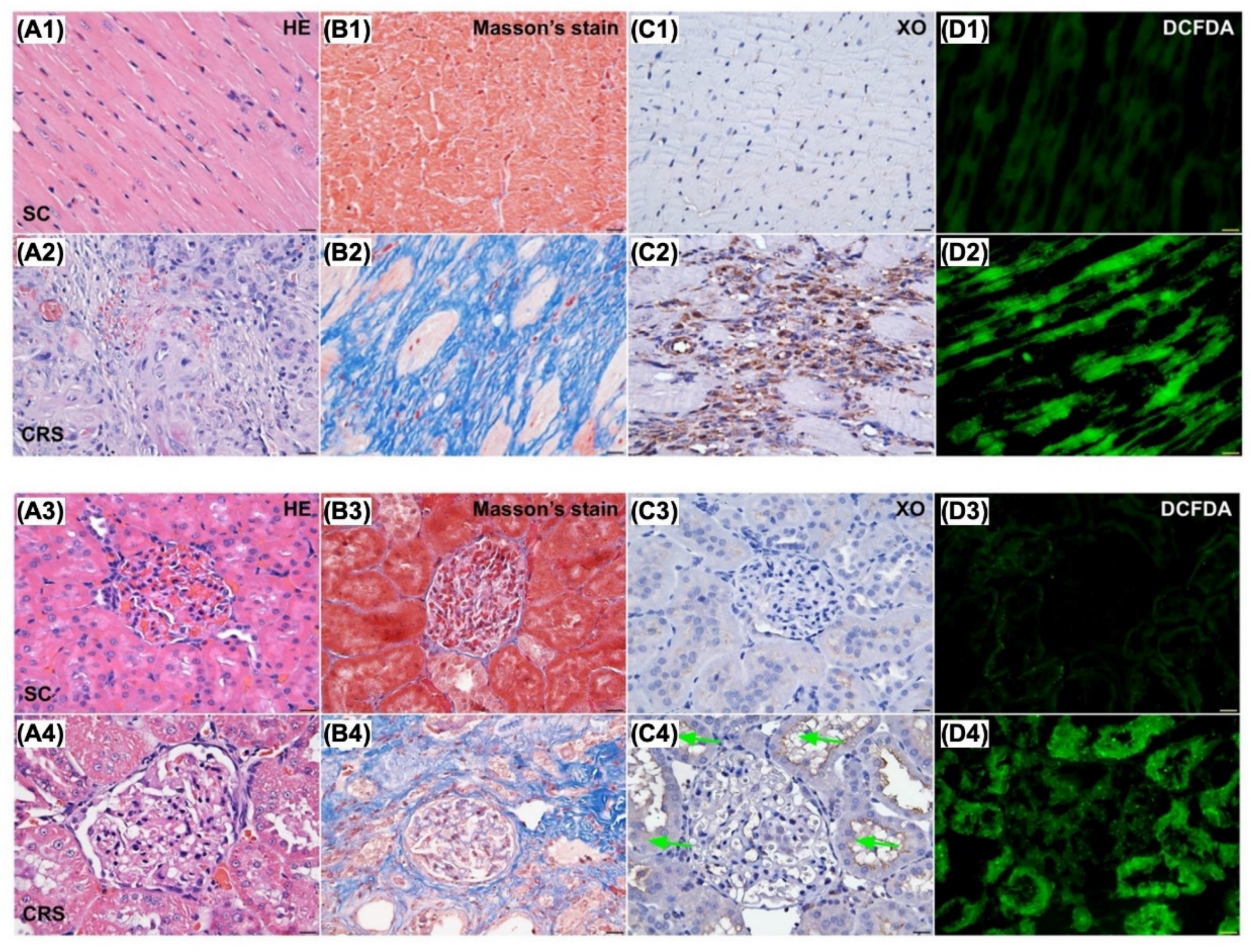
** Microscopic findings (400x) for identification of histopathological change, fibrosis, XO activity and ROS in LV myocardium and kidney parenchyma. Upper panel of histopathological findings in LV myocardium: A1 & A2)** Microscopic finding (100x) of H.E. stain demonstrated the histopathological changes, including lost cardiomyocytes, inflammatory cell infiltration (gray color) and disorganized of myocardial fiber architecture were notably increased in CRS animal (A2) than in SC animal (A1). Scale bar in right lower corner represents 100µm. **B1 & B2)** Microscopic finding (200x) of Masson's trichrome stain showed the fibrotic area (blue color) was remarkably increased in CRS (B1) than that of SC (B2). Scale bar in right lower corner represents 50µm. **C1 & C2)** Microscopic finding (400x) of immunohistochemical (IHC) stain showed that the XO expression (gray color) was markedly increased in CRS (C1) than that of SC (C2). Scale bar in right lower corner represents 20µm. **D1 & D2)** Microscopic finding (400x) of immunofluorescent (IF) stain showed that the fluorescent intensity (i.e., H_2_DCFDA staining) (green color) were markedly increased in CRS (D1) than that of SC (D2). Scale bar in right lower corner represents 20µm. SC = sham control; CRS = cardiorenal syndrome; XO = xanthine oxidase. **Lower panel of histopathological findings in kidney parenchyma: A3 & A4)** Microscopic finding (200x) of H.E. stain demonstrated the kidney injury scores, including loss of brush border in renal tubules, tubular necrosis, tubular dilatation, and dilatation of Bowman's capsule, were notably higher in CRS (A3) than in SC (A4). Scale bar in right lower corner represents 50µm. **B3 & B4)** Microscopic finding (200x) of Masson's trichrome stain showed the fibrotic area (blue color) were remarkably increased in CRS (B3) than that of SC (B4). Scale bar in right lower corner represents 50µm. **C3 & C4)** Microscopic finding (400x) of IHC stain showed that the XO expression (gray color), predominantly localized in renal tubules (green arrows), was markedly increased in CRS (C3) than that of SC (C4). Scale bar in right lower corner represents 20µm. **D3 & D4)** Microscopic finding (400x) of IF microscope showed that the fluorescent intensity (i.e., H_2_DCFDA staining) (green color) was markedly increased in CRS than that of SC. Scale bar in right lower corner represents 20µm. SC = sham control; CRS = cardiorenal syndrome; XO = xanthine oxidase; LV = left ventricular.

**Figure 6:**
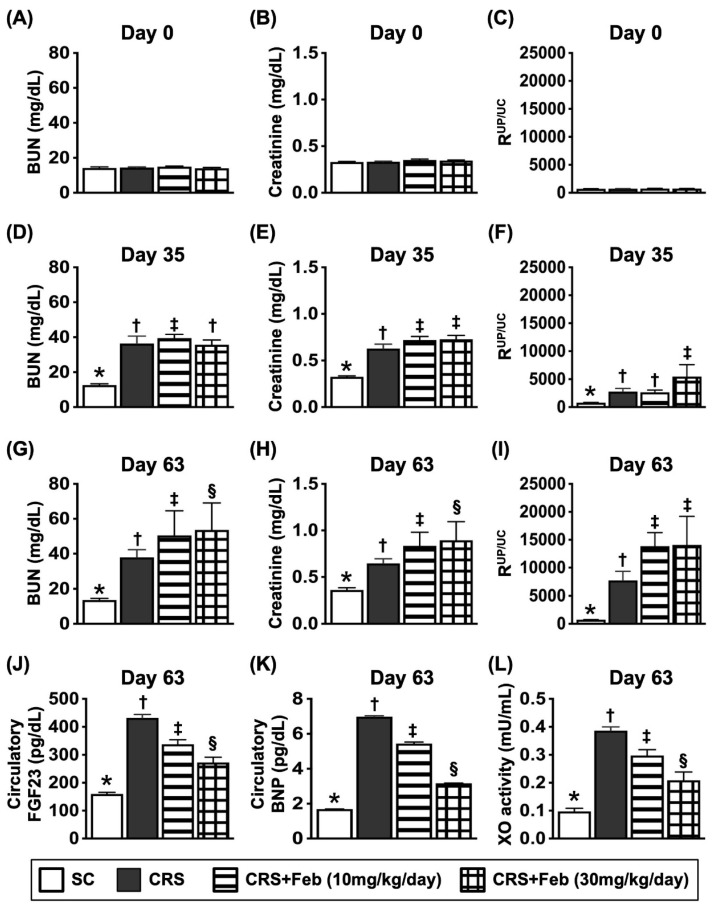
** The time courses of circulatory BUN and creatinine levels and R^Up/Uc^, and circulating levels of FGF23, BNP and XO activity by day 63 after CRS induction. A)** Circulatory level of blood urea nitrogen (BUN) by day 0, *P*>0.5. **B)** Circulatory level of creatinine by day 0, *P*>0.5. **C)** The ratio of urine protein to urine creatinine (R^Up/Uc^) by day 0, *P*>0.5. **D)** Circulatory level of BUN by day 35, * vs. †, *P*<0.0001. **E)** Circulating level of creatinine, * vs. †, *P*<0.0001. **F)** The R^Up/Uc^ by day 35, * vs. †, *P*<0.0001. **G)** Circulatory level of BUN by day 60, * vs. other groups with different symbols (†, ‡), *P*<0.0001. **H)** Circulatory level of creatinine by day 60, * vs. other groups with different symbols (†, ‡), *P*<0.0001. **I)** The R^Up/Uc^ by day 60, * vs. other groups with different symbols (†, ‡, §), *P*<0.0001. **J)** circulating level of fibroblast growth factor 23 (FGF23) by day 60, * vs. other groups with different symbols (†, ‡, §), *P*<0.0001. **K)** Circulatory level of brain natriuretic peptide (BNP) by day 60, * vs. other groups with different symbols (†, ‡, §), *P*<0.0001. **L)** Circulatory level of xanthine oxidase by day 60, * vs. other groups with different symbols (†, ‡, §), *P*<0.0001. All statistical analyses were performed by one-way ANOVA, followed by Bonferroni multiple comparison post hoc test (n=6-8) for each group). Symbols (*, †, ‡, §) indicate significance (at 0.05 level). Feb = febuxostat; CRS = cardiorenal syndrome.

When looked at the tissue levels of XO activity, ROS, fibrosis, and destructive severity of kidney and heart, we found that these parameters in both kidney and LV myocardium were notably higher in CRS group than in SC group (Figure 5). Accordingly, the results from Figures 1 to 6 encouraged us to conduct a complete CRS animal model to evaluate the effect of Feb therapy on protecting kidney and heart against the CRS-induced damage.

### The time courses of circulatory BUN and creatinine levels and R^Up/Uc^, circulating levels of FGF23, BNP and XO activity by day 63 after CRS induction (Figure 6)

The baseline levels of BUN, creatinine and R^Up/Uc^ showed no difference among the groups. However, by day 35 after CRS induction, these three parameters were significantly lower in group 1 (SC) than in groups 2 (CRS only), 3 [CRS + Feb (10 mg/kg/day)] and 4 [CRS + Feb (30 mg/kg/day)], but they showed no difference among groups 2, 3 and 4. Additionally, by days 63 after CRS induction, these parameters were lowest in group 1, highest in group 2 and notably increased in group 3 than in groups 4. Furthermore, by day 63 after CRS induction, the circulating levels of fibroblast growth factor 23 (FGF23) (i.e., acts as an inhibitor of renal phosphate reabsorption), brain natriuretic peptide (BNP) and XO, displayed an identical pattern of day-63 creatinine level among the groups.

### Serial changes of LVEF and RARI, and mortality rate by day 63 after CRS induction (Figure 7)

By day 0, the renal artery restrictive index (RARI) and LVEF did not differ among the four groups. On the other hand, by day 35 after CRS induction, the RARI was significantly lower in group 1 than in groups 2, 3 and 4, but it showed no difference among the latter three groups. Additionally, by day 63 after CRS induction this parameter was lowest in group 1, highest in group 2 and significantly higher in group 3 than in group 4, whereas the LVEF showed an opposite manner of RARI at the time points of days 35 and 63 among the groups.

A total of 83 animals were utilized in the present study. The mortality rate within 35 days, i.e., at the time interval of complete CRS induction prior to animal grouping, was 22.9% (19/83). The remaining of 64 animals were categorized into group 1 (n=9), group 2 (n=21), group 3 (n=16), and group 4 (n=18), respectively. By the end of the study period, the mortality rate was 0 % (0/9) in group 1, 42.9% (9/21) in group 2, 12.5% in group 3 and 22.2% in group 4, respectively. Analytical result showed the mortality rate was significantly higher in group 2 than in other groups, but it revealed no significant difference among the groups 1, 3 and 4.

### Impact of Feb therapy on regulating mitochondrial biogenesis signalings, oxidative stress and antioxidants in LV myocardium and kidney by day 63 after CRS induction (Figure 8)

To elucidate the therapeutic impact of Feb on the expressions of mitochondrial biogenesis signaling, oxidative stress and antioxidants in LV myocardium and kidney, the Western blot analysis was conducted in the present study. The result showed that the protein expressions of p-AMPK, SIRT1, and PGC-1α, three mitochondrial biogenesis biomarkers were highest in group 1, lowest in group 2 and significantly higher in group 4 than in group 3, whereas the protein expression of p-DRP1, an indicator of mitochondrial damage displayed an opposite pattern of PGC-1α. Additionally, the protein expression of NOX-2, NOX-4, and XO, three indices of oxidative stress displayed an opposite pattern of PGC-1α, whereas the protein expression of catalase and SOD1 catalase, two indicators of antioxidant also exhibited an opposite pattern of PGC-1α among the groups, suggesting due to intrinsic response to oxidative stress stimulation that could be notably neutralized by Feb treatment. Furthermore, the protein expressions of CHAC1 and CHAC2, two crucial factors for regulating the cellular ROS homeostasis, exhibited an opposite pattern of PGC-1α among the groups.

### Impact of Feb therapy on downregulating the fibrosis and inflammation in LV myocardium and kidney by day 63 after CRS induction (Figure 9)

To verify whether Feb treatment also effectively suppressed the fibrosis and inflammation, the Western blot analysis was conducted in the present study. The result showed that the protein expressions of p-Smad3 and TGF-β, two indices of fibrosis, and the protein expressions of p-NF-κB and IL-1β, two indices of inflammatory reaction, were lowest in group 1, highest in group 2 and significantly higher in group 3 than group 4.

### Cellular expressions of fibrosis, oxidative stress, inflammation and CHAC1 in LV myocardium and kidney by day 63 after CRS induction (Figures 10 to 12)

**Figure 10:**
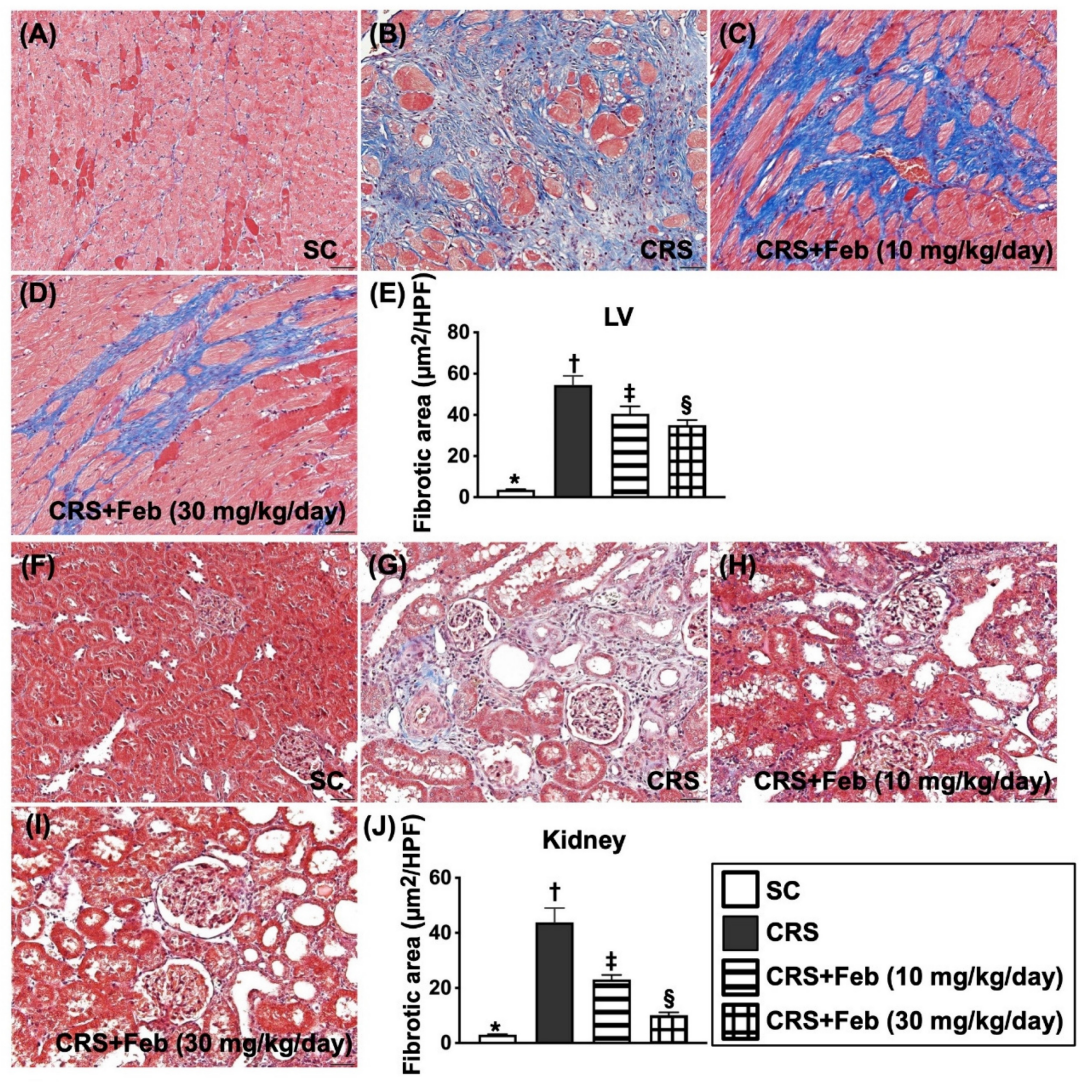
** Cellular expressions of fibrosis in LV myocardium and kidney by day 63 after CRS induction. A to D)** Illustrating the microscopic finding (200x) of Masson's trichrome stain for identification of fibrotic area in LV myocardium (blue color). **E)** Analytical result of fibrotic area, * vs. other groups with different symbols (†, ‡, §), *P*<0.0001. **F to I)** Illustrating the microscopic finding (200x) of Masson's trichrome stain for identification of fibrotic area in kidney parenchyma (blue color). **J)** Analytical result of fibrotic area, * vs. other groups with different symbols (†, ‡, §), *P*<0.0001. Scale bar in right lower corner represents 50µm. All statistical analyses were performed by one-way ANOVA, followed by Bonferroni multiple comparison post hoc test (n=6) for each group). Symbols (*, †, ‡, §) indicate significance (at 0.05 level). Feb = febuxostat; CRS = cardiorenal syndrome; LV = left ventricular.

**Figure 11:**
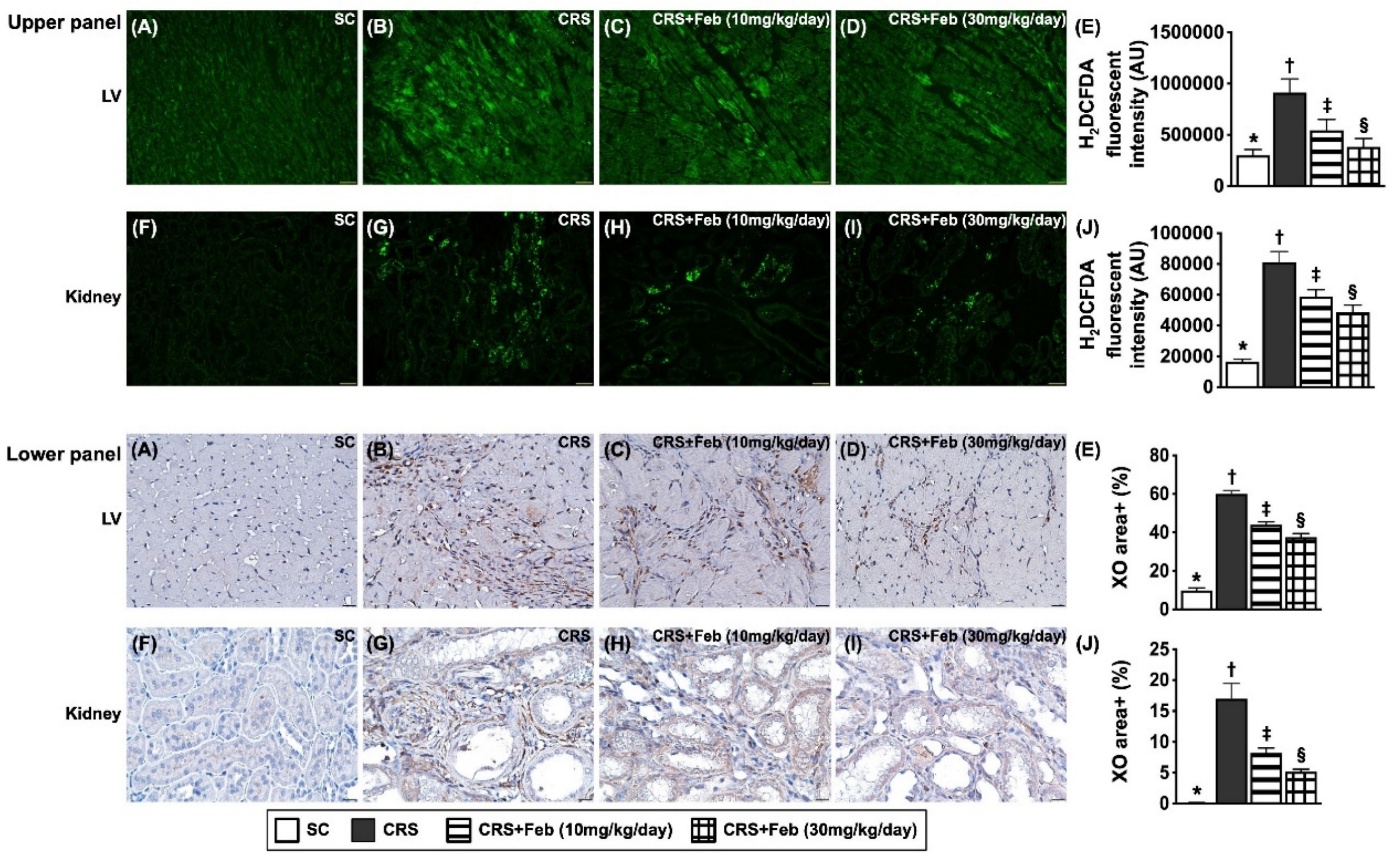
** Cellular expressions of reactive oxygen species (ROS) and xanthine oxidase (XO) in LV myocardium and kidney by day 63 after CRS induction. Upper panel: A to D)** Immunofluorescent (IF) microscopic finding (200x) for identification of cellular expression of ROS in LV myocardium (green color). **E)** Analytical result of mean fluorescent intensity (i.e., by H_2_DCFDA stain), * vs. other groups with different symbols (†, ‡, §), *P*<0.0001. **F to I)** The IF microscopic finding (200x) for identification of cellular expression of ROS in kidney parenchyma (green color). **J)** Analytical result of mean fluorescent intensity (i.e., by H_2_DCFDA stain), * vs. other groups with different symbols (†, ‡, §), *P*<0.0001. Scale bar in right lower corner represents 50µm. **Lower panel: A to D)** Illustrating the microscopic finding (400x) of immunohistochemical (IHC) stain for identification of cellular expression of XO in LV myocardium (gray color). **E)** Analytical result of XO expression score, * vs. other groups with different symbols (†, ‡, §), *P*<0.0001. **F to I)** Illustrating the microscopic finding (400x) of IHC stain for identification of cellular expression of XO in kidney parenchyma (gray color). **J)** Analytical result of XO expression score, * vs. other groups with different symbols (†, ‡, §), *P*<0.0001. Scale bar in right lower corner represents 20µm. All statistical analyses were performed by one-way ANOVA, followed by Bonferroni multiple comparison post hoc test (n=6) for each group). Symbols (*, †, ‡, §) indicate significance (at 0.05 level). Feb = febuxostat; CRS = cardiorenal syndrome; LV = left ventricular.

**Figure 12:**
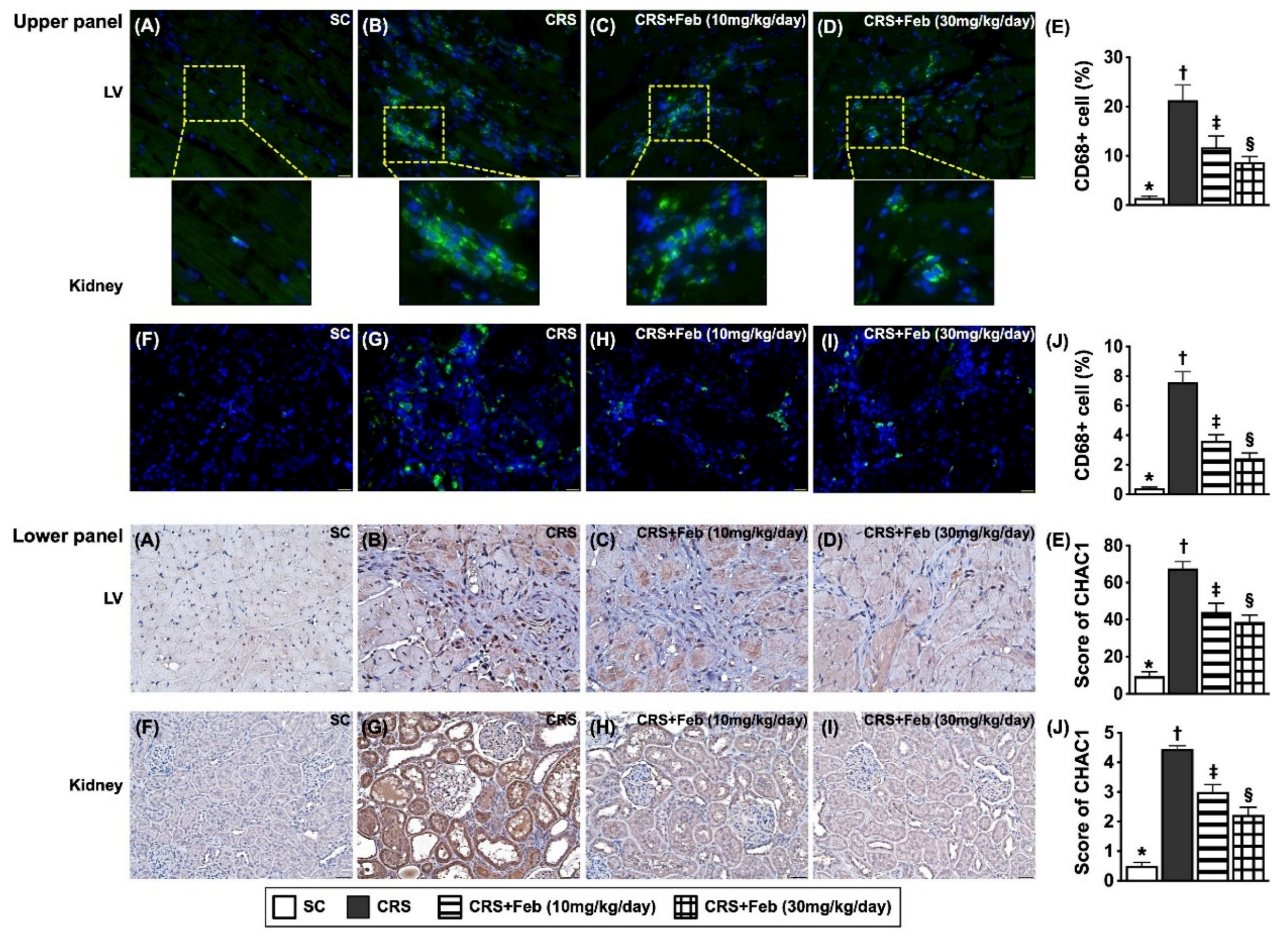
** Cellular expressions of inflammation and CHAC1 in LV myocardium and kidney by day 63 after CRS induction. Upper panel: A to D)** Illustrating the microscopic finding (400x) of immunofluorescent (IF) stain for identification of cellular expression of CD68 in LV myocardium (green color). **E)** Analytical result of number of CD68 expression (%), * vs. other groups with different symbols (†, ‡, §), *P*<0.0001. **F to I)** Illustrating the microscopic finding (400x) of IF stain for identification of cellular expression of CD68 in kidney parenchyma (green color). **J)** Analytical result of number of CD68 expression (%), * vs. other groups with different symbols (†, ‡, §), *P*<0.0001. **Lower panel: A to D)** Illustrating the microscopic finding (400x) of immunohistochemical (IHC) stain for identification of cellular expression of CHAC1 in LV myocardium (gray color). **E)** Analytical result of CHAC1 expression (%), * vs. other groups with different symbols (†, ‡, §), *P*<0.0001. **F to I)** Illustrating the microscopic finding (400x) of IHC stain for identification of cellular expression of CHAC1 in kidney parenchyma (gray color). **J)** Analytical result of CHAC1 expression score, * vs. other groups with different symbols (†, ‡, §), *P*<0.0001. Scale bar in right lower corner represents 20µm. All statistical analyses were performed by one-way ANOVA, followed by Bonferroni multiple comparison post hoc test (n=6) for each group). Symbols (*, †, ‡, §) indicate significance (at 0.05 level). Feb = febuxostat; CRS = cardiorenal syndrome; LV = left ventricular.

Microscopic finding demonstrated the cellular expressions of fibrosis (Fig. 10), H_2_DCFDA (Fig. 11) and XO (Fig. 11), two indicators of oxidative stress, CD68 (Fig. 12), an indicator of inflammation, and CHAC1 (Fig. 12), an enzyme that degrades the glutathione, resulting in upregulating ROS, were highest in group 2, lowest in group 1 and significantly lower in group 4 than in group 3.

## Discussion

In the present study, we investigated the therapeutic impact of Feb on CRS rodents yielded some specific clinical implications. First, Feb treatment significantly suppressed oxidative stress, fibrosis and inflammatory signalings via alleviating the mitochondrial ROS and XO activity and restoring the mitochondrial biogenesis signaling. Second, Feb treatment remarkably improved the renal and LV function and the renal artery compliance (i.e., reduced the renal artery resistance) in CRS rodent. Third, Feb treatment notably improved the survival rate in CRS rodent.

Interestingly, XO has been identified to never hide its identity involving in upregulating the oxidative stress in setting of heart failure, chronic kidney disease as well as in a variety of pathophysiological circumstances 8-12, 15-17, resulting in damage of myocardium, renal tubular cells/podocytes, kidney parenchyma, endothelial cells and mitochondria 13, ultimately causing renal failure and failing of myocardium through increased renal interstitial and myocardial fibrosis 9, 14, 15. When looked at the *in vitro* study, we found that p-Cresol, i.e., a uremic toxic substance, played a key role on enhancing oxidative stress, inflammatory reaction, DNA damage, mitochondrial dysfunctional and cell apoptosis as well as remarkably damage of podocyte components/renal tubular cells. When further deeply looked at the results of *in vitro* study, we found that p-Cresol was the prime culprit to markedly elevate the level of XO in podocytes and renal tubular cells. Intriguingly, the above-mentioned molecular perturbations were substantially repressed by Feb treatment or silencing the XO in renal tubular cells. Our findings, in addition to being comparable with the findings from previous studies 8-17, highlighted that XO was the fundamental requirements for over production of oxidative stress/ROS.

When looked at the *in vitro* studies, we observed that the therapeutic effect of Feb was not inferior to silencing the XO gene in renal tubular cells undergoing p-Cresol stimulation. These findings encouraged to us design a pilot study to delineate the correlation between XO activity and kidney/heart functions. Intriguingly, the results of the pilot study showed that (1) XO activity remarkably elevated and significantly positive correlated to heart and kidney dysfunction in rat CRS model by day 42 after CRS induction and (2) the kidney injury score and fibrosis, XO activity and generation of ROS in LV myocardium and kidney parenchyma were substantially augmented in CRS animals as compared with the SC groups. Thus, the *in vitro* and pilot studies motivated that we should conduct a more complete CRS animal model study to keenly investigated the suitable dose of Feb and the underlying mechanism of Feb on protecting the kidney and heart in CRS rodents.

Undoubtedly, the mortality, morbidity and unfavorable long-term outcome are substantially increased in CRS than those of merely one organ disorder 25-27. One important finding in the *in vivo* study was that the 63-day mortality rate was significantly increased in CRS animals as compared to that of the SC group. Our finding strengthened the findings of the previous studies 25-27. Of distinctive finding was that Feb treatment significantly reduced the mortality rate in CRS animals, highlighting that Feb treatment may pose as a therapeutic potential for those of CRS, especially when these patients associated with hyperuricemia.

Our previous study identified that not only the renal function but also the heart function was remarkably impaired in CRS animals 28. The most important finding in the present study was that as compared to the SC group, the renal function and LVEF were substantially impaired, whereas the creatinine level, proteinuria and RARI were remarkably increased in CRS animals. However, these parameters were substantially reversed by low dose (i.e., 10 mg/kg) and further substantially reversed by high dose (30 mg/kg) of Feb treatment. Our finding, in addition to reinforcing the finding of our previous study 28, encourage the use of optimal dose of Feb in clinical setting of CRS patients.

We designed to extensively evaluate not only in image studies, urine examination, or tissue specimen but also in circulatory biomarkers. An essential finding in the present study was that at the end of study period, the circulating levels of FGF23 (i.e., an inhibitor of renal phosphate reabsorption), BNP (a heart failure marker) and XO (an oxidative stress expressed in circulation) were significantly increased CRS animals than in SC animals, implicating that CRS could also be reliably evaluated for the severity of its clinical presentation by these circulatory biomarkers.

Upregulations of inflammation, oxidative stress and fibrosis were commonly found in CRS or CKD setting 23, 28, 29. In the present study, when looked at the heart and kidney parenchymal specimen, we found that cellular and protein levels of fibrosis, oxidative stress/ROS, XO activity and inflammatory reaction were significantly enhanced in CRS animals than in the SC animals. In this way, our findings, in addition to corroborating with the findings of our previous studies 23, 28, 29, could at least in part, explain why the heart and kidney functions were deteriorated and the proteinuria was augmented in the CRS animals.

Importantly, these parameters were significantly reversed in low dose and more significantly reversed in high dose of Feb treatment, whereas the antioxidant biomarkers were markedly increased by low dose and further remarkably increased by high dose of Feb treatment. These could, once again, partially explain why the kidney and heart function was great preserved and the mortality rate was notably reduced in CRS rat after receiving Feb treatment.

**Figure 13:**
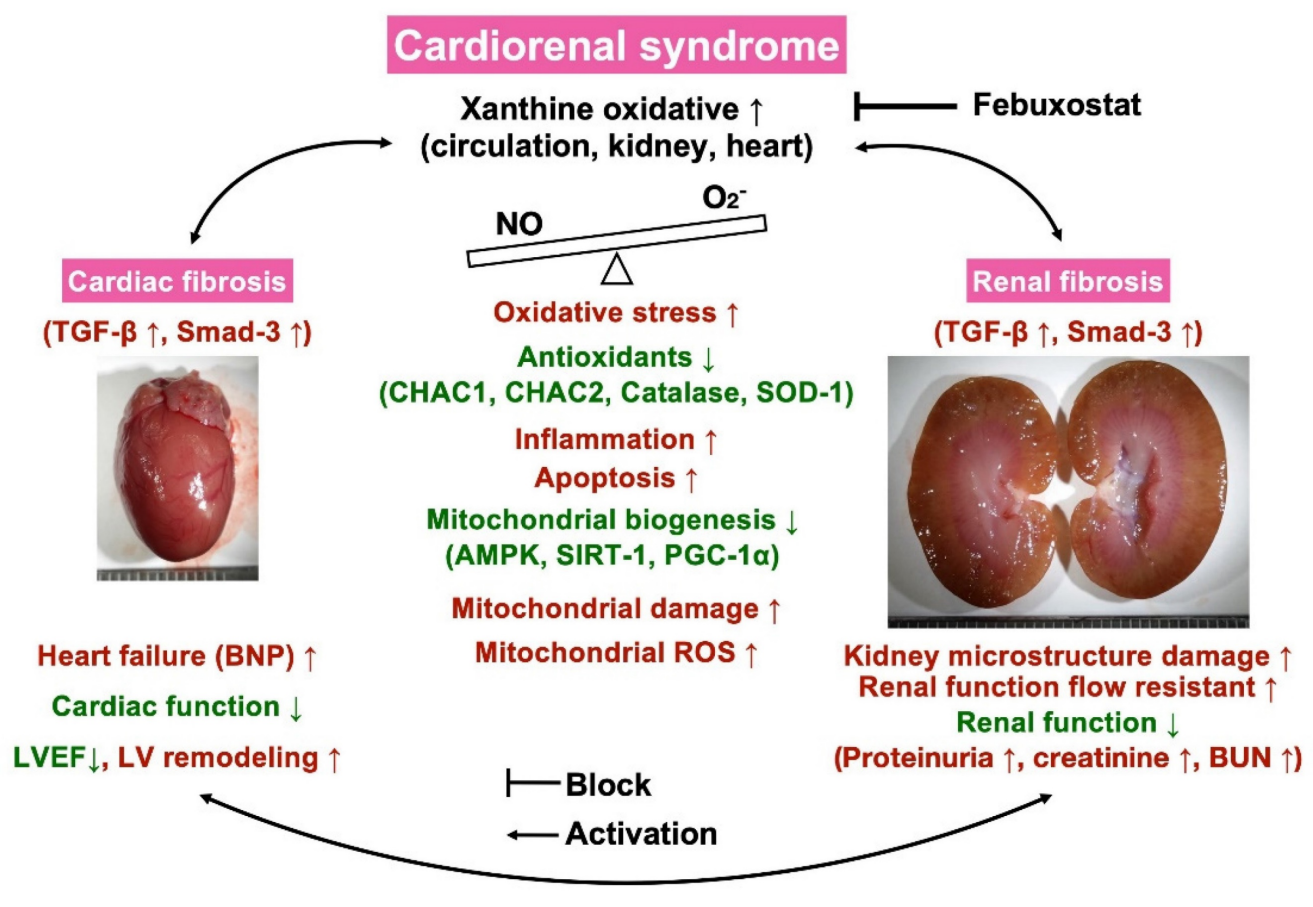
** Illustrasting the underlying mechanims of XO indcued cardiorenal syndrome and the impact of Feb treatment on protecting the heart and kidney organs in setting of CRS.** NO = nitric oxide; CRS = cardiorenal syndrome; LVEF = left ventricular ejection fraction; ROS = reactive oxygen species; Feb = Febuxostat.

Certainly, scientists and readers would be much interested in what is the underlying mechanism for the Feb treatment offering promising impact on reducing the mortality rate and proteinuria and preserving the heart and kidney functions in setting of CRS. Based on our extensive works, we could schematically propose (referred to Figure 13) that CRS elicited vigorously oxidative stress responses that were not only local (i.e., in myocardial and kidney tissues) but also systemic expressions as well as not only expressed in cellular but also expressed in molecular levels, which in turn comprehensively damaged the microstructural integrity of heart and kidney parenchyma, ultimately caused deterioration of heart and kidney function and unfavorably outcomes. On the other hand, Feb treatment markedly attenuated oxidative stress through suppressing XO expression and upregulating mitochondrial biogenesis signaling resulting in redox homeostasis and protecting the myocardium and renal parenchyma against oxidative stress damage.

### Study limitations

There are some limitations in the present study. First, although the underlying mechanisms of oxidative stress causing deteriorations of heart and kidney functions in CRS setting were schematically illustrated in Figure 13 thoroughly our extensive works, we could not exclude the exact mechanism of CRS to cause the heart and kidney functions would be more complicated than the result of our findings. Second, although our findings were attractive and promising, we did not know whether the effect Feb treatment could be extrapolated into our daily clinical practice with a comparable outcome as in our preclinical study.

In conclusion, our finding demonstrated that Feb treatment effectively preserved the heart and kidney functions and improved the outcomes predominantly via downregulating the generation of oxidative stress and upregulating the mitochondrial biogenesis signaling.

## Supplementary Material

Supplementary figure.

## Figures and Tables

**Figure 7:**
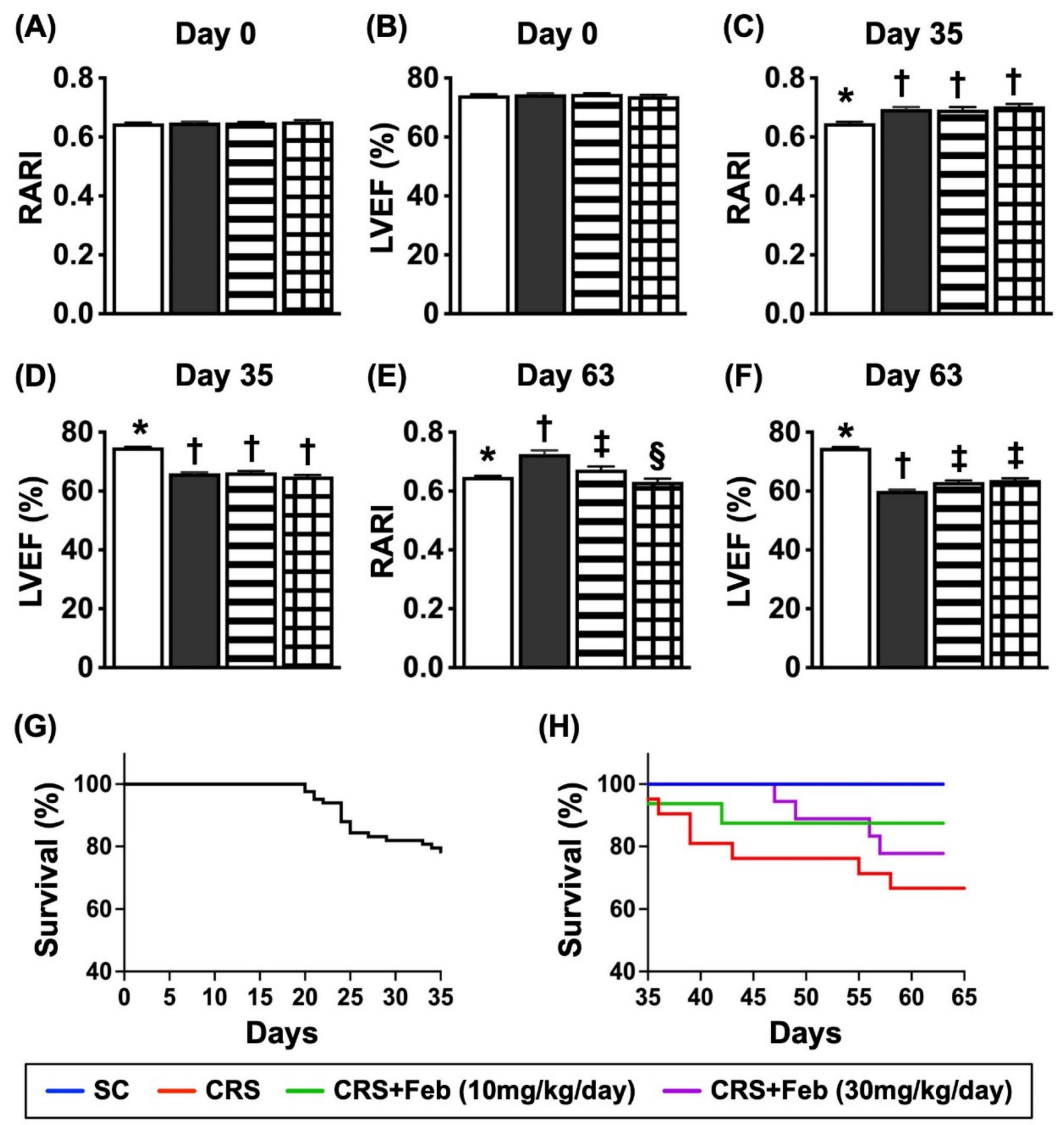
** Serial changes of LVEF and RARI, and mortality rate by day 63 after CRS induction. A)** Analytical result of renal artery restrictive index RARI at day 0, p>0.5. **B)** Analytical result of LVEF at day 0, *P*>0.5. **C)** Analytical result of RARI by day 35, * vs. †, *P*<0.0001. **D)** Analytical result of LVEF by day 35, * vs. †, *P*<0.0001. **E)** Analytical result of RARI by day 60, * vs. other groups with different symbols (†, ‡, §), *P*<0.0001. **F)** Analytical result of LVEF by day 60, * vs. other groups with different symbols (†, ‡), *P*<0.0001. **G)** Showing the survival rate (i.e., 77.1%) within the day 35 after CRS induction prior to grouping. **H)** Showing the accumulated survival rate from days 36 to 63 after CRS induction among the groups, * vs. *, *P*=0.029. All statistical analyses were performed by one-way ANOVA, followed by Bonferroni multiple comparison post hoc test (n=9-21) for each group). Symbols (*, †, ‡, §) indicate significance (at 0.05 level). Feb = febuxostat; CRS = cardiorenal syndrome. LVEF = left ventricular ejection fraction; RARI = renal artery resistive index.

**Figure 8:**
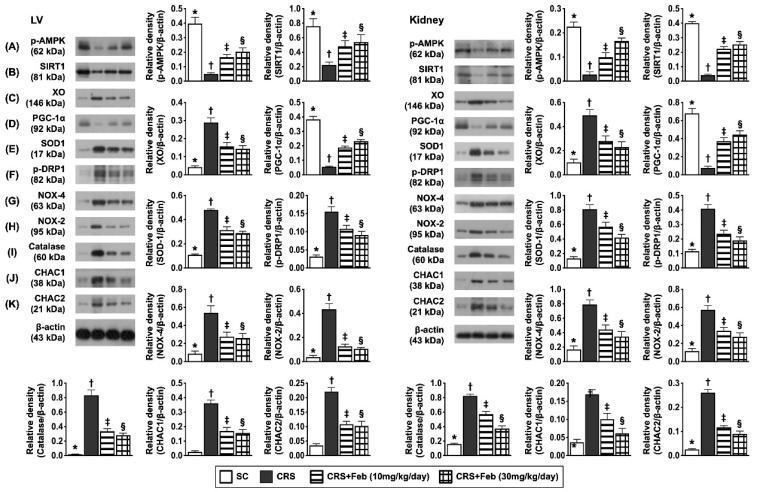
** Impact of Feb therapy on regulating mitochondrial biogenesis signalings, oxidative stress and antioxidants in LV myocardium (Upper panel) and kidney (Lower panel) tissues by day 63 after CRS induction. Left panel (LV myocardium) and Right panel (kidney): A)** Protein expression of phosphorylated (p)-AMPK, * vs. other groups with different symbols (†, ‡, §), *P*<0.0001. **B)** Protein expression of SIRT1, * vs. other groups with different symbols (†, ‡, §),* P*<0.0001. **C)** Protein expression of xanthine oxidase (XO), * vs. other groups with different symbols (†, ‡, §), *P*<0.0001. **D)** Protein expression of PGC-1α, * vs. other groups with different symbols (†, ‡, §), *P*<0.0001. **E)** Protein expression of SOD1, * vs. other groups with different symbols (†, ‡, §), *P*<0.0001. **F)** Protein expression of p-DRP1, * vs. other groups with different symbols (†, ‡, §), *P*<0.0001. **G)** Protein expression of NOX-4, * vs. other groups with different symbols (†, ‡, §), *P*<0.0001. **H)** Protein expression of NOX-2, * vs. other groups with different symbols (†, ‡, §), *P*<0.0001. **I)** Protein expression of catalase, * vs. other groups with different symbols (†, ‡, §), *P*<0.0001. **J)** Protein expression of ChaC glutathione specific gamma-glutamylcyclotransferase 1 (CHAC1), * vs. other groups with different symbols (†, ‡, §), *P*<0.0001. **K)** Protein expression of CHAC2, * vs. other groups with different symbols (†, ‡, §), *P*<0.0001. All statistical analyses were performed by one-way ANOVA, followed by Bonferroni multiple comparison post hoc test (n=6) for each group). Symbols (*, †, ‡, §) indicate significance (at 0.05 level). Feb = febuxostat; CRS = cardiorenal syndrome; LV = left ventricular.

**Figure 9:**
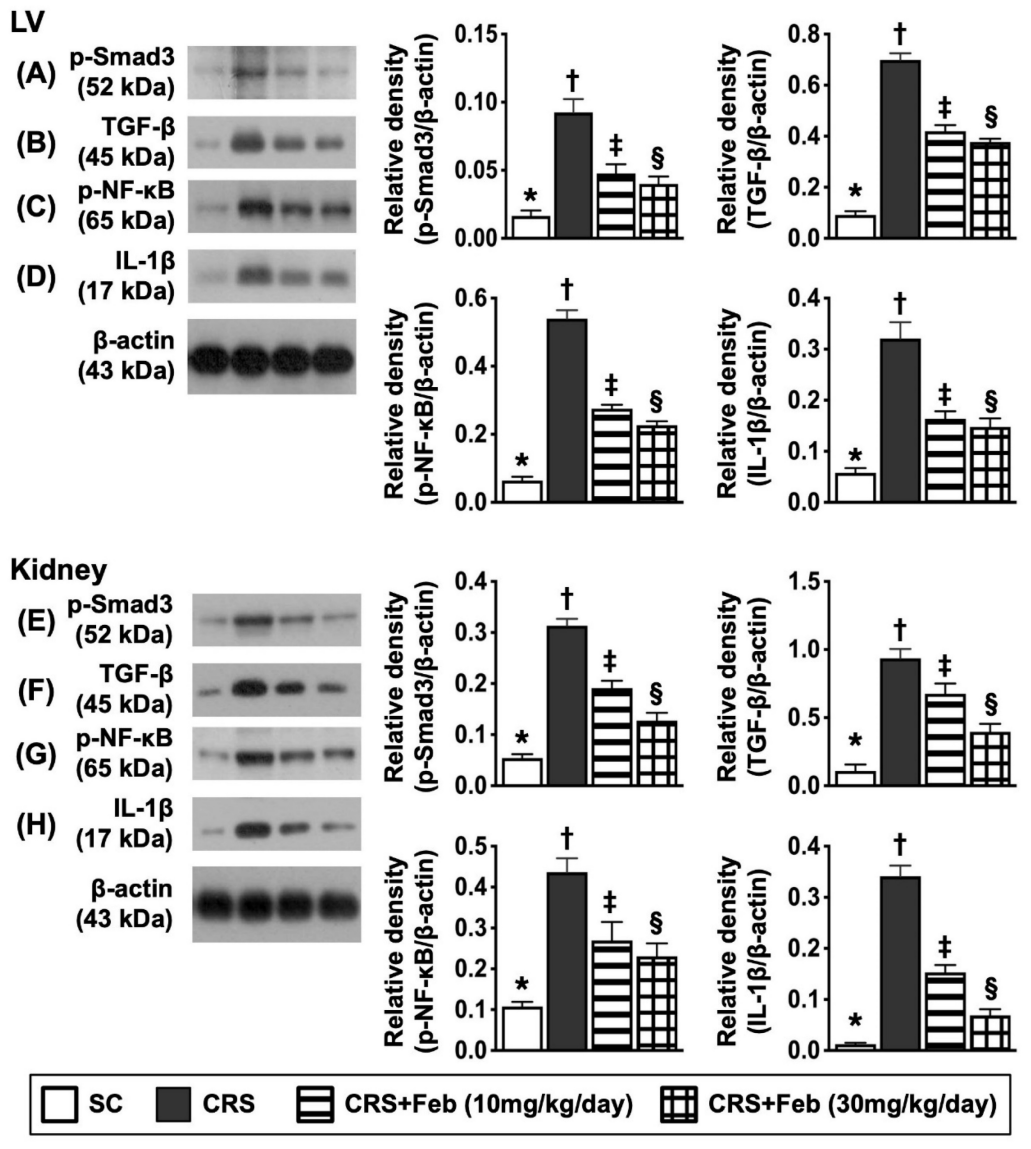
** Impact of Feb therapy on downregulating the fibrosis and inflammation in LV myocardium and kidney by day 63 after CRS induction. Upper panel (LV myocardium): A)** Protein expression of p-Smad3, * vs. other groups with different symbols (†, ‡, §), *P*<0.0001. **B)** Protein expression of TGF-β, * vs. other groups with different symbols (†, ‡, §),* P*<0.0001. **C)** Protein expression of phosphorylated (p)-nuclear factor (NF)-κB, * vs. other groups with different symbols (†, ‡, §), *P*<0.0001. **D)** Protein expression of interleukin (IL)-1β, * vs. other groups with different symbols (†, ‡, §),* P*<0.0001. **Lower panel (kidney): E)** Protein expression of p-Smad3, * vs. other groups with different symbols (†, ‡, §), *P*<0.0001. **F)** Protein expression of TGF-β, * vs. other groups with different symbols (†, ‡, §),* P*<0.0001. **G)** Protein expression of p-NF-κB, * vs. other groups with different symbols (†, ‡, §), *P*<0.0001. **H)** Protein expression of interleukin (IL)-1β, * vs. other groups with different symbols (†, ‡, §), *P*<0.0001. All statistical analyses were performed by one-way ANOVA, followed by Bonferroni multiple comparison post hoc test (n=6) for each group). Symbols (*, †, ‡, §) indicate significance (at 0.05 level). Feb = febuxostat; CRS = cardiorenal syndrome; LV = left ventricular.

## Abbreviations

ATP: Adenosine triphosphate
BNP: brain natriuretic peptide
BUN: blood urine nitrogen
CKD: chronic kidney disease
CRS: Cardiorenal syndrome
CVD: cardiovascular disease
DCM: dilated cardiomyopathy
DMSO: Dimethyl sulfoxide
DNA: deoxyribonucleic acid
DOX: doxorubicin
ECL: enhanced chemiluminescence
eGFR: estimated glomerular filtration rate
ELISA: Enzyme-linked immunosorbent assay
Feb: febuxostat
H&E stain: haematoxylin and eosin stain
HF: heart failure
HPFs: High-power fields
IF: immunofluorescent
IHC: immunohistochemistry
LDV: lowest diastolic blood velocity
LV: left ventricular
LVEDd: LV end-diastolic diameter
LVESd: LV end-systolic diameter
LVEF: left-ventricular-ejection-fraction
MTT: 3-(4,5-Dimethylthiazol-2-yl)-2,5-diphenyltetrazolium bromide
NO: Nitric oxide
NOX: NADPH oxidase
PI: propidium iodide
PSV: peak systolic blood velocity
PVDF: polyvinylidene difluoride
RARI: renal artery resistive index
ROS: reactive oxygen species
RT-PCR: Real-time polymerase chain reaction
SDS-PAGE: sodium dodecyl sulfate-Polyacrylamide gel electrophoresis
XDH: xanthine dehydrogenase
XO: xanthine oxidase
XOR: Xanthine oxidoreductase
