# Supplementary material for: Febuxostat therapy improved the outcomes of cardiorenal syndrome rodent through alleviating xanthine oxidase-induced oxidative stress and mitochondrial dysfunction
Source: Int J Biol Sci. 2025 Feb 10;21(4):1749–66. doi: 10.7150/ijbs.99194 (PMC11844282; doi:10.7150/ijbs.99194)
Supplement: Supplementary file 1 — Supplementary figure. [file ijbsv21p1749s1.pdf]

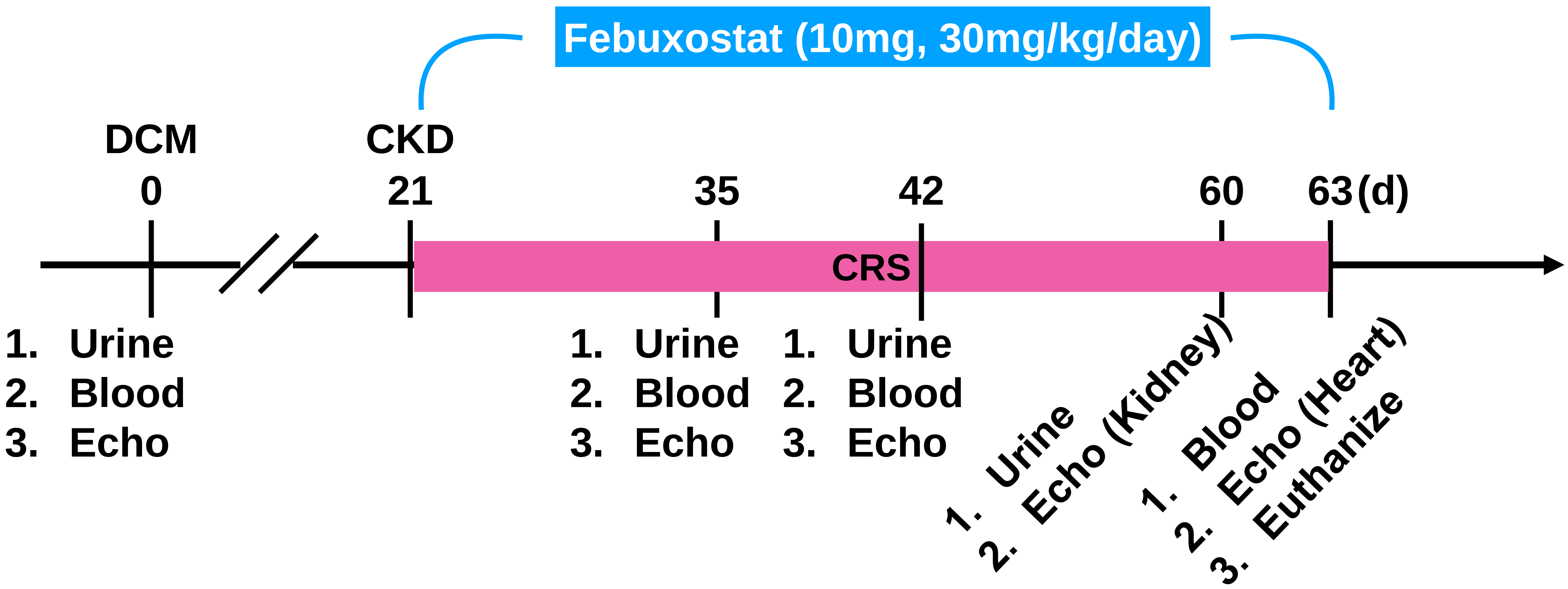

Supplementary Figure 1. Time points of CRS induction and febuxostat therapy

Supplementary Figure 1 schematically illustrated the time points of CRS induction, blood sampling, the transthoracic echocardiographic examination, therapeutic intervention and end of the study period. CRS = cardiorenal syndrome; DCM = dilated cardiomyopathy; CKD = chronic kidney disease; 2-D echo = 2-dimensional echocardiography.
